# The clinical implication of CD45RA^+^ naïve T cells and CD45RO^+^ memory T cells in advanced pancreatic cancer: a proxy for tumor biology and outcome prediction

**DOI:** 10.1002/cam4.1988

**Published:** 2019-02-14

**Authors:** Junjie Hang, Junjie Huang, Siyuan Zhou, Lixia Wu, Yingwei Zhu, Lina Zhu, Hanyu Zhou, Kequn Xu, Hua Jiang, Xuguang Yang

**Affiliations:** ^1^ Department of Oncology, Changzhou No.2 People’s Hospital Affiliated Hospital of Nanjing Medical University Changzhou Jiangsu China; ^2^ JC School of Public Health and Primary Care Chinese University of Hong Kong Hong Kong China; ^3^ Department of Oncology Shanghai JingAn District ZhaBei Central Hospital Shanghai China; ^4^ State Key Laboratory for Oncogenes and Related Genes, Department of Oncology, Shanghai Cancer Institute, Renji Hospital, School of Medicine Shanghai Jiao Tong University Shanghai China; ^5^ Cancer Institute, Longhua Hospital Shanghai University of Traditional Chinese Medicine Shanghai China

**Keywords:** advanced pancreatic cancer, memory T cells, naïve T cells, prognostic value, progression prediction

## Abstract

Naïve and memory T cells play a pivotal role in solid tumor pathogenesis but their role in pancreatic cancer progression remains elusive. Thus, we aimed to investigate their clinical potential in advanced pancreatic cancer (APC). Flow cytometry was performed to evaluate the level of baseline peripheral naïve and memory T cells from 137 APC patients before receiving first‐line chemotherapy. Interrelationships between naïve, memory T cells and clinicopathological variables were evaluated using Pearson’s correlation. The prognostic impact of naïve and memory T cells were assessed by Kaplan‐Meier analysis and Cox regression. The correlation between naïve/memory T cells and tumor progression was investigated by Student’s *t* test. CD4^+^ naïve/memory ratio showed close correlations with hemoglobin, red blood cell (RBC), absolute neutrophil count (ANC) and platelet while CD8^+^ naïve/memory ratio was correlated with hemoglobin, RBC and CEA. Higher baseline lever of CD4^+^CD45RO^+^/CD4^+^ was correlated with better overall survival (OS) (*P* = 0.036). Patients with CD4^+^ naïve/memory ratio ≥0.36 had a poorer OS than those with CD4^+^ naïve/memory ratio <0.36 (*P* = 0.021). In addition, CD4^+^ naïve/memory ratio showed independent prognostic impact (HR 1.427, 95% CI 1.033‐1.973, *P* = 0.031). Furthermore, poorer clinical response was correlated with higher level of CD8^+^ naïve/memory ratio after the third cycle of chemotherapy (*P* = 0.01). Besides, patients with a lower level of CD8^+^ naïve/memory ratio had longer progression‐free survival (PFS) (*P* = 0.028). We propose CD4^+^ naïve/memory ratio as a novel prognostic biomarker for APC. In addition, CD8^+^ naïve/memory ratio can be a candidate marker for predicting PFS and the change of its level may reflect the progression of APC.

## 1 INTRODUCTION

Pancreatic cancer is a highly lethal malignancy with an overall 5‐year survival of approximately 6%.^1^ In a world Health Organization (WHO) survey of global cancer statistics in 2012, it ranks the 7th leading cause of cancer‐related mortality.^2^ Despite the progress in the strategies of diagnosis and therapy, the prognosis for pancreatic cancer patients remains extremely dismal.^3^ Therefore, it is of great significance to identify novel biomarkers to improve outcome for patients with pancreatic cancer by assisting precise decision‐making and allowing individual risk stratification.

Dysregulation in cancer patient's immune system is one of hallmarks of cancer.^4^ Considerable evidence reveals that the host immune reaction plays a pivotal role in both inhibition and promotion of cancer growth.^5^ This pivotal immune function involves both naïve T cells and memory T cells. Naïve T cells express CD45RA and are usually functionally quiescent^6^; but in responses to stimuli, they may produce a high level of chemokines, such as CXCL8, which mediates neutrophil migration to a tumor and promotes tumor growth.^7^ In contrast, memory T cells are of the CD45RO^+^ phenotype,^8^ and secrete IFN‐γ, CCL4, XCL1 and other cytokines to kill tumor cells directly or indirectly.^9^, ^10^

In previous studies, changes in both naïve and memory T cells in solid tumors were shown to have prognostic impact, but their prognostic role in pancreatic cancer stay elusive.^11^, ^12^, ^13^, ^14^, ^15^, ^16^ For example, in 2011, Fogar et al found that pancreatic cancer cells could induce activated CD4 T cells (CD69^+^), but not CD4 memory (CD45RO), naïve (CD45RA), and regulatory (CD25) subsets.^17^ Bae J et al found that XBP1 peptide‐specific cytotoxic T lymphocytes (XBP1‐CTL), which were enriched with CD45RO^+^ memory CTL, showed high expression of critical T cell markers, such as CD28, with anti‐tumor activities in pancreatic cancer, breast cancer, and colon cancer cells.^18^ Recently, Farren MR et al described that metastatic pancreatic cancer patients with a greater proportion of memory T cells had a significantly longer OS, but they did not illustrate the role of memory T cells in this setting.^19^ The aim of the current study was to investigate the clinical implication of naïve and memory T cells in pancreatic cancer.

## 2 METHODS

### 2.1 Patients and sample collection

A total of 137 patients with APC (ICD‐10, codes C25) were enrolled at the Changzhou No.2 People's Hospital and Shanghai JingAn District ZhaBei Central Hospital. Inclusion criteria were: (a) pathologically confirmed pancreatic ductal adenocarcinoma, either by surgical resection or needle biopsy; (b) locally advanced unresectable or metastasis disease diagnosed by computed tomography (CT) or magnetic resonance imaging (MRI); (c) receiving at least three cycles of first‐line chemotherapy after the first diagnosis. Peripheral blood samples was collected in all patients prior to the first cycle of first‐line chemotherapy, and also in 12 patients before the second, third, and after the third cycles of chemotherapy. In addition, peripheral blood samples were also collected in 10 healthy controls. Patients’ and controls’ written informed consents and approval from the Ethics Committees of Changzhou No.2 People's Hospital were obtained for the use of these clinical materials. The methods were carried out in accordance with the approved guidelines.

### 2.2 Flow cytometry for lymphocyte subset analyses

**Figure 1:**
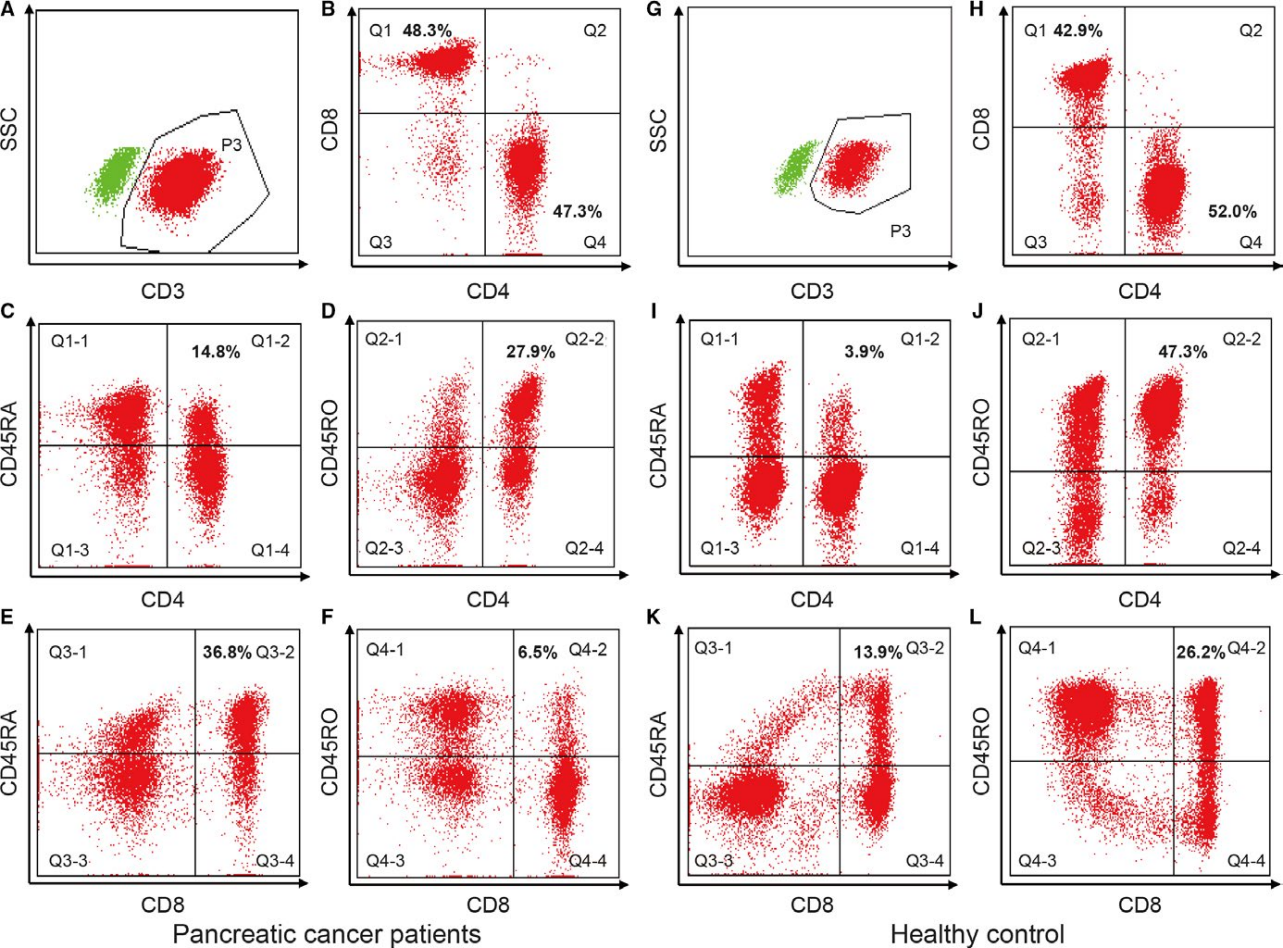
Flow cytometric analyses of peripheral blood lymphocytes by FACSCanto (BD Bioscience). The figures are representative of a pancreatic cancer patient and a healthy control. For pancreatic cancer patient, CD3^+^ T cells are assessed in region P3 (A); CD4^+^ and CD8^+^ T cells are evaluated in region Q1 and Q4 (B); CD4^+^ naïve T cells are detected in region Q1‐2 (C); CD4^+^ memory T cells are detected in region Q2‐2 (D); CD8^+^ naïve T cells are detected in region Q3‐2 (E); CD8^+^ memory T cells are detected in region Q4‐2 (F). For the healthy control, CD3^+^ T cells are assessed in region P3 (G); CD4^+^ and CD8^+^ T cells are evaluated in region Q1 and Q4 (H); CD4^+^ naïve T cells are detected in region Q1‐2 (I); CD4^+^ memory T cells are detected in region Q2‐2 (J); CD8^+^ naïve T cells are detected in region Q3‐2 (K); CD8^+^ memory T cells are detected in region Q4‐2 (L)

A routine clinical flow cytometry test protocol was followed for evaluation of lymphocytes in the peripheral blood. Briefly, 100uL whole blood was added into a tube and cells were blocked with 2.5 µg of Human BD Fc Block for 10 min and then stained with antibodies in PBS containing 0.1% (wt/vol) BSA and 0.1% NaN3. The following antibodies were purchased from BD Biosciences (San Jose, CA, USA): anti‐CD4 (RPA‐T4), anti‐CD8 (RPA‐T8), anti‐CD45RA (HI100), anti‐CD45RO (UCHL1). Then, 2 mL 1X RBC Lysis Buffer (Biolegend, 420301) were added into the samples. After incubation for 10‐15 min at room temperature, cells were collected by centrifugation at 270 *g* for 5 min and resuspended in FACS staining buffer. The FCM data were acquired on a BD FACSCanto and data were analyzed by using BD FACSDiva flow cytometry analysis software. The representative images of CD4^+^/CD8^+^ naïve and memory T cells in APC and healthy control were shown in Figure 1.

### 2.3 Statistical analysis

The statistical analyses were conducted with SPSS statistical software (version 21.0; SPSS Inc, IBM, Armonk, NY, USA). The Pearson's correlation coefficient was used to evaluate the strength of the linear relationships among naïve, memory T cells and clinicopathological variables. The Kaplan‐Meier analysis was conducted to assess the prognosis and the log rank test was used to compare the survival between groups. In Kaplan‐Meier analysis, the cut‐off value of CD4^+^ or CD8^+^ naïve/memory T cells and their ratios were identified as the median level of these variables. The Cox regression model was used to investigate the independent prognostic variables. The correlation of naïve/memory T cells in response to first‐line chemotherapy was assessed with the two‐tailed Student's *t* test. Progression‐free survival is defined as the time from the initiation of the first‐line chemotherapy to the earlier of death or disease progression. In addition, Kaplan‐Meier analysis was used to evaluate the PFS in different groups. Two‐sided *P* < 0.05 was considered statistically significant in all tests.

## 3 RESULTS

### 3.1 Patient characteristics

The majority of patients were male (63.5%) and the median age was 60 (range 36‐81) years. Most of patients have distant metastasis (67.9%) at initial diagnosis. The detailed clinicopathological characteristics of patients were demonstrated in Table 1.

**Table 1:** Baseline clinicopathological characteristics of patients with APC

Valuables	Category	Characteristics
Gender	Male	87 (63.5%)
	Female	50 (36.5%)
Age	Median (range)	60 (36‐81)
ECOG PS	0	14 (10.2%)
	1	108 (78.8%)
	2	15 (10.9%)
Primary tumor site	Head and neck	58 (42.3%)
	Body and tail	79 (57.7%)
Distant metastasis	No	44 (32.1%)
	Yes	93 (67.9%)
Hb (g/L)	Median (range)	123 (41‐157)
RBC (*10^12^/L)	Median (range)	4.08 (1.24‐5.40)
ALC (*10^9^/L)	Median (range)	1.38 (0.28‐3.76)
AMC (*10^9^/L)	Median (range)	0.40 (0.11‐3.50)
ANC (*10^9^/L)	Median (range)	3.98 (1.12‐26.30)
PLT (*10^9^/L)	Median (range)	179 (45‐763)
CA19‐9 log value (U/mL)	Median (range)	2.92 (‐0.10‐3.32)
CEA (ng/mL)	Median (range)	6.4 (0.07‐1065.0)
AST (IU/L)	Median (range)	26.0 (10.6‐356.0)
ALT (IU/L)	Median (range)	22.2 (6.0‐305.3)
CD4^+^CD45RA^+^/CD4^+^ (%)	Median (range)	25.5 (2.4‐72.9)
CD4^+^CD45RO^+^/CD4^+^ (%)	Median (range)	70.6 (11.1‐96.4)
CD4^+^CD45RA^+^/CD45RO^+^	Median (range)	0.36 (0.02‐6.57)
CD8^+^CD45RA^+^/CD8^+^ (%)	Median (range)	52.4 (24.2‐91.7)
CD8^+^CD45RO^+^/CD8^+^ (%)	Median (range)	41.1 (6.6‐72.6)
CD8^+^CD45RA^+^/CD45RO^+^	Median (range)	1.28 (0.33‐12.91)

### 3.2 Circulating naïve/memory T cells and their correlation

**Figure 2:**
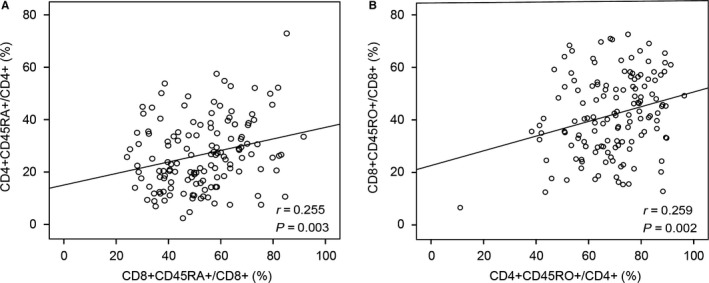
Correlation between CD4^+^ and CD8^+^ naïve/memory T cells. There was a positive correlation between the percentage of CD4^+^ and CD8^+^ naïve T cells (A) or memory T cells (B)

The distribution of circulating CD4^+^ or CD8^+^ naïve/memory T cells was shown in Table 1. Figure 2 showed that there was a significant correlation between the percent of CD4^+^ naïve T cells and CD8^+^ naïve T cells (*r* = 0.255, *P* = 0.003). The percent of CD4^+^ memory T cells was also positively correlated with the percent of CD8^+^ memory T cells (*r* = 0.259, *P* = 0.002).

### 3.3 Interrelationships between clinicopathological variables

We analyzed the interrelationships between CD4^+^ and CD8^+^ naïve/memory T cells and other clinicopathological variables of APC (Table 2). CD8^+^CD45RA^+^/CD8^+^ and CD8^+^CD45RO^+^/CD8^+^ was positively correlated with hemoglobin. CD4^+^ naïve/memory ratio showed close correlations with hemoglobin (*r* = −0.235, *P* = 0.006), RBC (*r* = −0.202, *P* = 0.019), ANC (*r* = 0.182, *P* = 0.033), and PLT (*r* = 0.191, *P* = 0.025). In contrast, CD8^+^ naïve/memory ratio was closely correlated with hemoglobin (*r* = −0.219, *P* = 0.010), RBC (*r* = −0.178, *P* = 0.038), and CEA (*r* = 0.195, *P* = 0.023). In addition, CD4^+^CD45RO^+^/CD4^+^ demonstrated a weak correlation with PLT (*r* = −0.173, *P* = 0.044). However, most of clinicopathological variables showed no significant correlation with CD4^+^CD45RA^+^/CD4^+^, CD4^+^CD45RO^+^/CD4^+^, CD8^+^CD45RA^+^/CD8^+^, and CD8^+^CD45RO^+^/CD8^+^.

**Table 2:** Interrelationship between clinicopathological variables

Characteristics	CD4^+^CD45RA^+^/CD4^+^ (%)	CD4^+^CD45RO^+^/CD4^+^ (%)	CD4^+^CD45RA^+^/CD45RO^+^	CD8^+^CD45RA^+^/CD8^+^ (%)	CD8^+^CD45RO^+^/CD8^+^ (%)	CD8^+^CD45RA^+^/CD45RO^+^
Age	NS	NS	NS	NS	NS	NS
Hb (g/L)	NS	NS	0.006	0.038	0.024	0.010
RBC (*10^12^/L)	NS	NS	0.019	NS	NS	0.038
ALC (*10^9^/L)	NS	NS	NS	NS	NS	NS
AMC (*10^9^/L)	NS	NS	NS	NS	NS	NS
ANC (*10^9^/L)	NS	NS	0.033	NS	NS	NS
PLT (*10^9^/L)	NS	0.044	0.025	NS	NS	NS
CA19‐9 log value	NS	NS	NS	NS	NS	NS
CEA (ng/mL)	NS	NS	NS	NS	NS	0.023
AST (IU/L)	NS	NS	NS	NS	NS	NS
ALT (IU/L)	NS	NS	NS	NS	NS	NS

### 3.4 Prognostic impact of naïve and memory T cells

**Figure 3:**
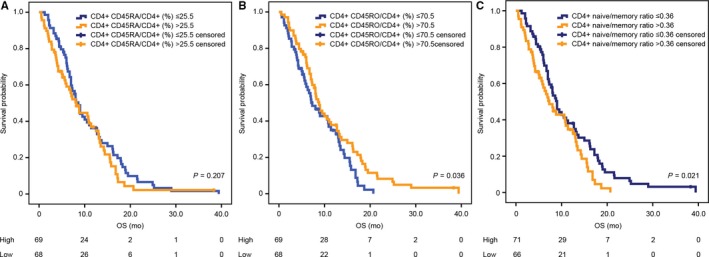
Kaplan‐Meier estimates of overall survival according to CD4^+^CD45RA^+^/CD4^+^ (%) (A), CD4^+^CD45RO^+^/CD4^+^ (%) (B), and CD4^+^ naïve/memory ratio (C)

Kaplan‐Meier analyses revealed that patients with CD4^+^CD45RO^+^/CD4^+^ (%) ≥70.5 seemed to have a better OS compared to those with CD4^+^CD45RO^+^/CD4^+^ (%) <70.5 (*P* = 0.036, Figure 3B). In addition, patients with CD4^+^ naïve/memory ratio ≥0.36 had a poorer OS compared to those with CD4^+^ naïve/memory ratio <0.36 (*P* = 0.021, Figure 3C). However, no significant difference in survival was found in high or low CD4^+^CD45RA^+^/CD4^+^ (%) group (Figure 3A). In addition, there was also no association between CD8^+^CD45RA^+^/CD8^+^ (%), CD8^+^CD45RO^+^/CD8^+^ (%), CD8^+^ naïve/memory ratio and survival.

### 3.5 Multivariate analysis: independent prognostic value of CD4^+^ naïve/memory ratio

All significant demographic and clinicopathological factors, including Eastern Cooperative Oncology Group (ECOG PS), distant metastasis, carbohydrate antigen 19‐9 (CA19‐9) log value, CD4^+^ naïve/memory ratio and ANC, from the univariate analysis were incorporated into the multivariate Cox regression model (Table 3). The former four were independent prognostic factors. Among them, CD4^+^ naïve/memory ratio showed independent prognostic impact (HR 1.427, 95% CI 1.033‐1.973, *P* = 0.031).

**Table 3:** Univariate and multivariate analysis of prognostic factors for OS in patients with APC

Characteristics	Univariate analysis			Multivariate analysis		
	HR	95% CI	*P*‐value	HR	95% CI	*P*‐value
Age (years, range)	0.993	0.974‐1.013	0.504			
Gender						
Male	1.006	0.691‐1.465	0.973			
Female	Reference					
Performance status						
ECOG PS = 2	2.802	1.609‐4.880	<0.001	2.593	1.356‐4.957	0.004
ECOG PS = 0‐1	Reference			Reference		
Primary tumor site						
Head and neck	1.35	0.931‐1.956	0.113			
Body and tail	Reference					
Distant metastasis						
Yes	2.182	1.466‐3.247	<0.001	2.427	1.599‐3.685	<0.001
No	Reference			Reference		
CEA (ng/mL)	1.001	1.000‐1.002	0.054			
CA 19‐9 log‐value (U/mL)	1.251	1.023‐1.530	0.029	1.217	1.001‐1.481	0.049
Hemoglobin (g/L)	1.004	0.990‐1.017	0.593			
RBC (*10^12^/L)	1.239	0.862‐1.782	0.247			
ALC (*10^9^/L)	0.898	0.684‐1.178	0.436			
ANC (*10^9^/L)	1.084	1.032‐1.138	0.001	1.016	0.956‐1.080	0.612
AMC (*10^9^/L)	1.469	0.965‐2.236	0.073			
PLT (*10^9^/L)	1.000	0.998‐1.002	0.827			
ALT (IU/L)	1.001	0.997‐1.005	0.683			
AST (IU/L)	0.999	0.996‐1.003	0.660			
CD4^+^CD45RA^+^/CD4^+^ (%)	1.009	0.995‐1.023	0.210			
CD4^+^CD45RO^+^/CD4^+^ (%)	1.989	0.975‐1.003	0.116			
CD4^+^CD45RA^+^/CD45RO^+^	1.588	1.118‐2.257	0.010	1.427	1.033‐1.973	0.031
CD8^+^CD45RA^+^/CD8^+^ (%)	1.001	0.990‐1.013	0.823			
CD8^+^CD45RO^+^/CD8^+^ (%)	0.999	0.987‐1.011	0.889			
CD8^+^CD45RA^+^/CD45RO^+^	1.018	0.900‐1.152	0.778			

### 3.6 Dynamics of naïve/memory T cells and its correlation with first‐line treatment

**Figure 4:**
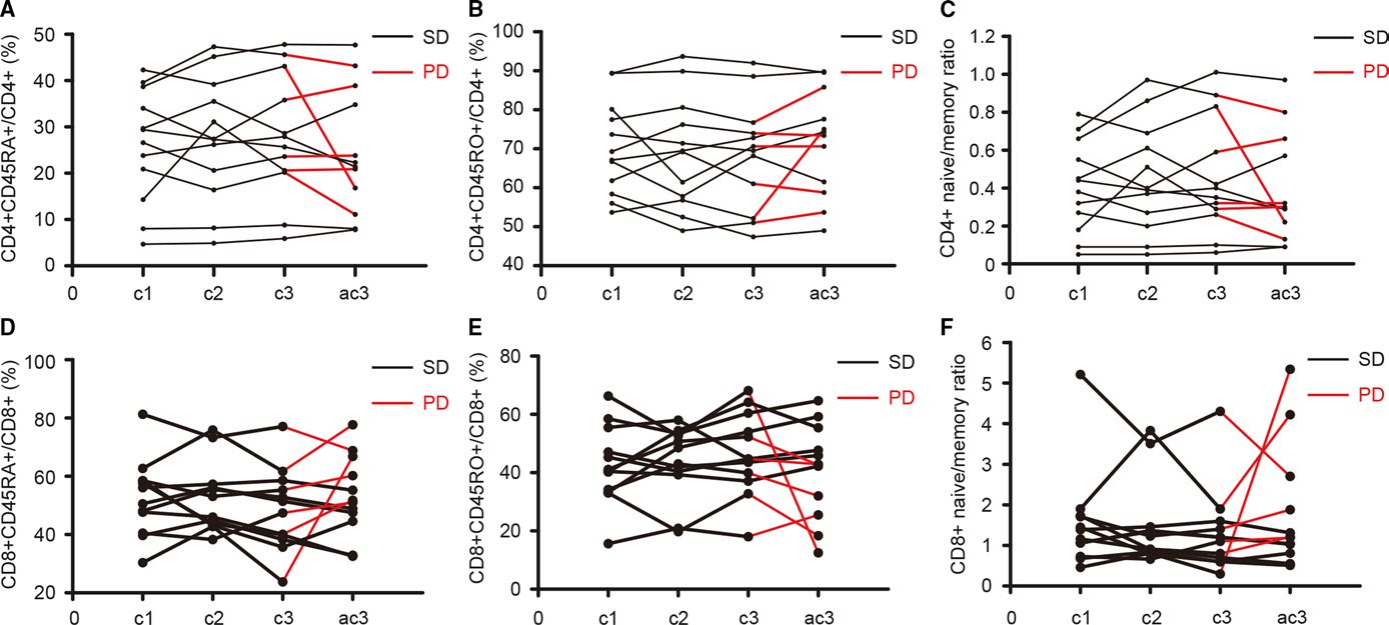
The change in percent of naïve/memory T cells in APC during first‐line treatment. Data of CD4^+^CD45RA^+^/CD4^+^ (A), CD4^+^CD45RO^+^/CD4^+^ (B), CD4^+^ naïve/memory ratio (C), CD8^+^CD45RA^+^/CD8^+^ (D), CD8^+^CD45RO^+^/CD8^+^ (E), and CD8^+^ naïve/memory ratio (F) are shown for samples taken before the first, second, and third cycle and after the third cycle of first‐line chemotherapy. Abbreviation: c1, before cycle 1; c2, before cycle 2; c3, before cycle 3; ac3, after cycle 3; SD, stable disease; PD, progressive disease

**Figure 5:**
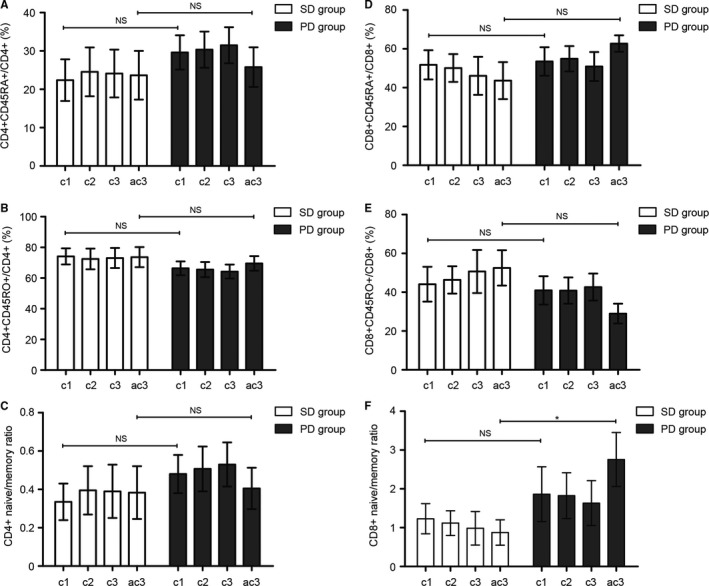
Correlation of percent of naïve/memory T cells with response to first‐line treatment. The change in CD4^+^CD45RA^+^/CD4^+^ (%) (A), CD4^+^CD45RO^+^/CD4^+^ (%) (B), CD4^+^ naïve/memory ratio (C), CD8^+^CD45RA^+^/CD8^+^ (%) (D), CD8^+^CD45RO^+^/CD8^+^ (%) (E) showed no difference between the SD group and PD group. The low level of CD8^+^ naïve/memory ratio was correlated with improved clinical response (SD vs PD: 0.87 ± 0.13 vs 2.76 ± 0.69, *P* = 0.01). The data are demonstrated as the mean ± SEM and the *P *values are determined by the Student’s *t* test (* SD compared with PD, *P* < 0.05). Abbreviation: c1, before cycle 1; c2, before cycle 2; c3, before cycle 3; ac3, after cycle 3; NS, not significant; SD, stable disease; PD, progressive disease

To investigate the correlation of percent of naïve/memory T cells with response to first‐line treatment, we collected the blood of 12 APC patients before the first, second and third cycle and after the third cycle of first‐line chemotherapy (Figure 4). All these patients were evaluated after the third cycle of first‐line chemotherapy by CT or MRI scans according to RECIST 1.1. Among them, six were evaluated as PD while another six were evaluated as SD. Figure 4 depicted the level of CD4^+^CD45RA^+^/CD4^+^ (%), CD4^+^CD45RO^+^/CD4^+^ (%), CD4^+^ naïve/memory ratio, CD8^+^CD45RA^+^/CD8^+^ (%), CD8^+^CD45RO^+^/CD8^+^ (%), and CD8^+^ naïve/memory ratio of these patients. The data were demonstrated as the mean ± SEM in Figure 5 and the *P *values were determined by the Student's *t* test. No significant difference was observed between the SD group and PD group in the level of CD4^+^CD45RA^+^/CD4^+^ (%), CD4^+^CD45RO^+^/CD4^+^ (%), CD4^+^ naïve/memory ratio, CD8^+^CD45RA^+^/CD8^+^ (%), CD8^+^CD45RO^+^/CD8^+^ (%), and CD8^+^ naïve/memory ratio before the first, second and third cycle of first‐line chemotherapy (Figure 5A‐E). However, poorer clinical response was correlated with the higher level of CD8^+^ naïve/memory ratio after the third cycle of first‐line chemotherapy (SD vs PD: 0.87 ± 0.13 vs 2.76 ± 0.69, *P* = 0.01, Figure 5F).

### 3.7 The correlation between naïve/memory T cells and PFS

**Figure 6:**
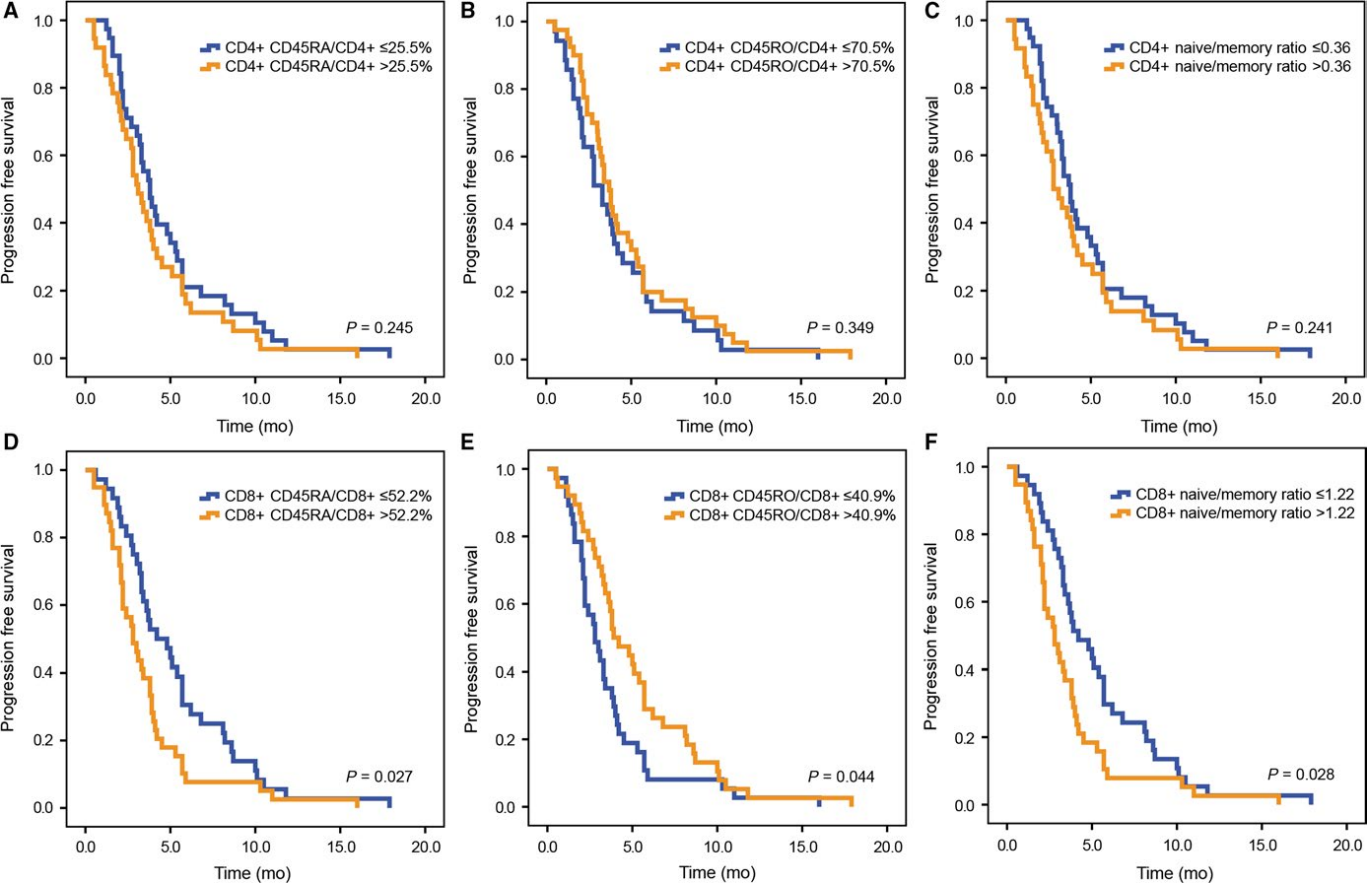
Kaplan‐Meier estimates of progression free survival according to CD4^+^CD45RA^+^/CD4^+^ (A), CD4^+^CD45RO^+^/CD4^+^ (B), CD4^+^ naïve/memory ratio (C), CD8^+^CD45RA^+^/CD8^+^ (D), CD8^+^CD45RO^+^/CD8^+^ (E), CD8^+^ naïve/memory ratio (F)

Patients with a low level of CD8^+^CD45RA^+^/CD8^+^ (%) had a better PFS than high level (median PFS 4.2 vs 2.8 months, *P* = 0.027, Figure 6D). In contrast, elevated CD8^+^CD45RO^+^/CD8^+^ (%) predicted better PFS (median PFS 3.9 vs 2.8 months, *P* = 0.044, Figure 6E). Besides, Kaplan‐Meier analysis revealed that patients with a lower level of CD8^+^ naïve/memory ratio had longer PFS (median PFS 4.2 vs 2.8 months, *P* = 0.028, Figure 6F). However, there was no significant difference between patients with low or high level of CD4^+^CD45RA^+^/CD4^+^ (%), CD4^+^CD45RO^+^/CD4^+^ (%), and CD4^+^ naïve/memory ratio (Figure 6A‐C).

## 4 DISCUSSION

Pancreatic cancer is an immunogenic tumor correlated with various kinds of immune/inflammatory cells. Among them, previous studies showed that both naïve T cells and memory T cells played a pivotal role in pathogenesis but their role in pancreatic cancer progression remains elusive.^7^ Thus, we try to investigate the clinical implication of CD4^+^/CD8^+^ naïve and memory T cells in APC patients. In this study, we found a novel association between the percent of CD4^+^ naïve T cells and CD8^+^ naïve T cells, CD4^+^ memory T cells, and CD8^+^ memory T cells. In addition, both CD4^+^ naïve/memory ratio and CD8^+^ naïve/memory ratio showed close correlations with hemoglobin and RBC. The baseline level of hemoglobin and RBC usually reflect the nutrition status of patients with APC and is found to be correlated with prognosis.^20^ As immunonutrition can suppress the inflammatory response, it is reasonable that there are positive correlation between hemoglobin, RBC, and naïve/memory ratio.^21^

As we described before, both naïve and memory T cells showed their prognostic impact in various tumors but have not been extensively explored in pancreatic cancer. In our study, we found there was no association between CD4^+^CD45RA^+^/CD4^+^, CD8^+^CD45RA^+^/CD8^+^, CD8^+^CD45RO^+^/CD8^+^, and CD8^+^ naïve/memory ratio and survival. However, higher baseline levers of CD4^+^CD45RO^+^/CD4^+^ and lower level of CD4^+^ naïve/memory ratio were positively correlated with better OS (*P* = 0.036 and 0.021, respectively). In addition, among these 6 immune parameters, only CD4^+^ naïve/memory ratio showed independent prognostic impact in multivariate analysis (HR 1.427, 95% CI 1.033‐1.973, *P* = 0.031). Intriguingly, Peng Yang et al also found similar result in nonsmall cell lung cancer that elevated CD4^+^ naïve/memory ratio, rather than the other five immune parameters, correlated with prolonged progression‐free survival (*P* = 0.013).^22^ These results suggested that the ratio, rather than absolute count of naïve and memory T cells was more suitable to reveal the systemic inflammatory response to cancer. This may be explained by the fact the absolute count of different phenotypes of T cells are also affected by other factors, such as age, to different extent.^8^ For instance, previous studies demonstrated that there was an increase in the number of circulating memory T cells, especially CD4^+^ memory T cells over time.^23^, ^24^ Evidence also showed that naïve T cells were negatively correlated or even decreased with age.^24^, ^25^

The efficacy and prediction of resistance to chemotherapy in patients with APC are essential to prolong PFS and further improve prognosis. Recent studies revealed that inflammatory cells might affect the component of the pancreatic stroma to affect the progression of pancreatic cancer. The levels of CD3, CD4, CD8, CD68, and CD206 expression in pancreatic cancer were found to be independently correlated with tumor recurrence by immunohistochemistry.^26^ In addition, during clinical management, chemotherapy was found to be associated with long‐term changes in different phenotypes of circulating lymphocyte levels.^11^, ^27^ In this study, we demonstrated that the low level of CD8^+^ naïve/memory ratio after the third cycle of first‐line chemotherapy was correlated with improved clinical response (SD vs PD: 0.87 ± 0.13 vs 2.76 ± 0.69, *P* = 0.01). Such phenomenon suggested that the induction or maintenance of CD8^+^ memory T cells and inhibition of CD8^+^ naïve T cells could be a novel treatment strategy to ensure the long‐term efficacy of chemotherapy. Besides, Kaplan‐Meier analysis revealed that patients with a lower level of CD8^+^ naïve/memory ratio had longer PFS than those with a higher level (median PFS 4.2 vs 2.8 months, *P* = 0.028). However, there was no significant difference between patients with low or high level of CD4^+^ naïve/memory ratio. This may be partly explained by the reason that CD8^+^ T cells, especially CD8^+^ T memory cells, can efficiently recognize cancer targets and eliminate cancer cells via IFN‐γ‐mediated direct effects on malignant cells while different phenotypes of CD4^+^ T cells (Th1, Th2, and Th17) showed both protumorigenic and antitumor effects.^5^ In the progression of pancreatic cancer, the number of CD8^+^ T cells will decrease distinctly and among CD8^+^ T cells, memory T cells have a shorter half‐life than naïve T cells (1‐12 months compared with 1‐8 years respectively).^28^ Thus, CD8^+^ naïve/memory ratio may reflect the progression of pancreatic cancer in a better way than other variables.

There are several limitations of this study. First, the sample size of the patients, in whom the change of percent of naïve/memory T cells during first‐line treatment was investigated, was not large enough to reach definitive conclusions, which calls for further larger‐scale studies. Second, there are three major subsets of memory T cells in the blood: stem cell memory T cells, central memory T cells (T_CM_) and effector memory T cells (T_EM_), which have different properties and capacities.^8^ Because of its design, our work cannot solve the question of difference in clinical impact of specific phenotype of memory T cells on pancreatic cancer. Third, an external validation cohort is still needed in the future to make the result more convincing.

In conclusion, we found that CD4^+^ naïve/memory ratio can be a novel prognostic biomarker for advanced pancreatic cancer. In addition, CD8^+^ naïve/memory ratio can be a candidate marker for predicting PFS and its change may reflect the progression of APC.

## CONFLICTS OF INTEREST

None.
